# Do you remember tuberculosis?

**DOI:** 10.3325/cmj.2021.62.101

**Published:** 2021-02

**Authors:** Charles H. Calisher

COVID-19, COVID-19, COVID-19! That is all we have been hearing about since February of last year, as though that is the only infectious disease that is occurring at the present time. Yes, this probably is the most startling pandemic of our lives. Yes, this newly recognized coronavirus causes all sorts of unusual and unexpected signs and symptoms in humans, strange pathogeneses, age-specific illnesses, high case-fatality rates in certain human populations, has racial biases, is found over a wide host range, has caused political chaos, economic impacts, media and Trumpian nonsenses, etc. Well, we are now in 2021 (We never changed the name to COVID-20, so I expect we won’t change it to COVID-21 or COVID-22; it might be best to just drop the number as this might take years to fully subside.) and this nightmare continues and unfortunately may continue until vaccines help to control the pandemic, then the epidemics, then the outbreaks, then the clusters of cases. After that, it is likely that humans will be inconvenienced and life-threatened by this virus for the foreseeable future.

So much interest has been focused on characterizing this virus, such as determining its geographic and host ranges, molecular peculiarities, immunological responses and antibody persistence, and many other traits, as well as potential treatment regimens, that the scientific literature is now inundated with papers summarizing studies by thousands of investigators (>150,000, according to one informal tally). After this cream has been skimmed from the milk, deeper investigations will be even more helpful. As this pandemic shrinks in size and intensity, we will, however, have to return to investigations of previously studied diseases, including some that have been with us for a long time and others that have been recognized relatively recently.

The World Health Organization (WHO, www.who.int) lists numerous bacterial, rickettsial, fungal, protozoan, and viral causes of disease (Table 1), some of which are commented on later in a few more details.

Tuberculosis, caused by *Mycobacterium tuberculosis* bacteria, was described in India in 1500 B.C. In 2017 there were an estimated 10.0 million new TB cases. In 2017 an estimated 1.3 million human immunodeficiency virus-negative people and 0.30 million human immunodeficiency virus-positive people died from TB.

Malaria, caused by plasmodium parasites, affected 228 million people, causing 405 000 deaths in 2017; disruptions of malaria prevention programs during the current pandemic have led to a surge in cases. Malaria will likely have killed more people in sub-Saharan Africa in 2020 than will have COVID-19 as the pandemic has caused disruptions of established public health initiatives and health care treatment protocols. There likely will have been up to 100 000 excess malaria deaths, primarily among children under the age of five, in Africa in 2020 due to COVID-19-related disruptions.

Several viral diseases may also be pointed out due to their public health risks. Acquired immunodeficiency syndrome (AIDS) is a chronic, potentially life-threatening condition caused by the human immunodeficiency virus. According to WHO, by the end of 2019 there were more than 38 million people in the world living with AIDS. Influenza is still a life-threating disease – WHO estimates that seasonal influenza may result in 290 000-650 000 deaths each year due to respiratory diseases alone. The same is with hepatitis B, estimated by WHO to cause about 780 000 deaths each year due to its consequences and complications, such as liver cirrhosis and liver cancer. Ebolaviruses cause, relatively speaking, not many cases but a frightening and highly pathogenic disease. Unfortunately, poliomyelitis is not yet completely eradicated. Hundreds of other bacterial, fungal, protozoan, or viral agents are known but seem to rarely cause illness and are not listed here. In addition, studies of antimicrobial resistance and host defense failures require attention.

COVID-19 is a disease that is preoccupying the scientific community and the public but it is just the latest infectious agent to do that. When an effective vaccine against COVID-19 is distributed and applied to humans, the recognition of COVID-19 will have provided funding for studies of other viruses, for immunologic studies, for improved preparedness, as well as for mortgages, wines, and travel to exotic lands.

Meanwhile, the diseases listed above, as well as others not yet recognized, will continue to impact humans and COVID-19 likely may not have taught us enough that is useful regarding what are already recognized as threats to human, wildlife, and livestock populations. In addition to the current and near future impact, COVID-19 has had and will have on health systems, it would be well for local, state, national, and international health systems to prepare for these changes. For example, cancer patients, whose essential and regular treatments are delayed or reduced because of changed priorities due to an influx of COVID-19 patients and attendant shortages of personnel, supplies, and equipment, would likely be devastatingly impacted. Community surveys for tuberculosis and treatment of those with evidence of tuberculosis might be curtailed, as might progress in further diagnosing and thereby reducing malaria in places where it occurs and in international travel, and otherwise reducing recognition of less commonplace diseases certainly would be the result of focusing preponderantly on COVID-19 to the exclusion of other diseases. This is not likely to occur, because sensible case-managers would not allow it, but it could at least lead to bias in treatment of patients with more ordinary, long-term, or unusual debilities.

These days, funding for diagnosis, treatment, and research is derived mostly from public resources (taxes), and it is public media that inform us of the “disease of the week” (or years, by such as COVID-19). Sooner or later (sooner we all hope), COVID-19 will be controlled and be seen as just another virus infection. Meanwhile, we must retain and even expand training for younger people, those who will comprise the next generation of diagnosticians, nurses, clinicians, researchers, and others who will help fight the next pandemics and help protect humanity. First priority is to ensure, by international agreement, that disease reporting (local to national, national to international) be rapid, honest, and complete, which has not been the case.

Nobel Laureate Joshua Lederberg said: “The single biggest threat to man’s continued dominance on the planet is the virus.” If he was correct, job openings for would-be virologists and associated others will soon be a growth industry but, if we ignore the prevention, treatment, and other studies of non-pandemic microbes, we might all soon be dead and leave the earth to cockroaches (order Blattodea)!

**Table 1:** The World Health Organization's list of bacterial, fungal, protozoan, and viral diseases

Bacterial diseases	Fungal diseases	Protozoan diseases	Viral diseases
actinomycosis, anthrax, boutonneuse fever, brucellosis, spondylitis, campylobacteriosis, Carrión disease, cat scratch disease, cervicitis, chancroid, chlamydia, lymphogranuloma venereum, cholera, clostridial infection, dysentery, shigellosis, epididymitis, erysipelothrix infection, glanders, gonorrhea, granuloma inguinale, Legionnaires’ disease, leprosy, leptospirosis, listeriosis, Lyme disease, melioidosis, nocardiosis, paratyphoid fever, pharyngitis, plague, bubonic plague, pneumonia, proctitis, pseudotuberculosis, psittacosis, Q fever, rat-bite fever, Reiter syndrome (reactive arthritis), relapsing fever, rheumatic fever, Rocky Mountain spotted fever, salmonellosis, scarlet fever, septicemia, Waterhouse-Friderichsen syndrome, shigellosis, streptobacillary fever, syphilis, bejel, gumma, yaws, tetanus, tonsillitis, toxic shock syndrome, trench fever, tuberculosis, scrofula, tularemia, typhoid fever, typhus, scrub typhus, urethritis, vaginitis, vesiculitis, whooping cough, and yersiniosis	aspergillosis, blastomycosis, candidiasis, thrush, chromoblastomycosis, coccidioidomycosis, cryptococcosis, histoplasmosis, pharyngitis, pneumonia, sporotrichosis, urethritis, and vaginitis	avian malaria, Chagas disease, coccidiosis, leishmaniasis, Oriental sore, malaria blackwater fever, sleeping sickness, toxoplasmosis, trichomoniasis, trypanosomiasis	acquired immunodeficiency arboviruses (including Japanese encephalitis virus, West Nile virus, Powassan virus and other tick-borne encephalitis viruses, eastern equine, western equine and Venezuelan equine encephalitis viruses, yellow fever virus, Zika virus, Rift Valley fever virus, chikungunya fever virus, Colorado tick fever virus, pappataci fevers (sandfly fevers), and dengue viruses), influenza, hepatitis B, ebolaviruses, bird influenza, Borna disease, distemper, dysentery, herpangina, herpes simplex, herpes zoster, swine flu, measles, mumps, pharyngitis, pneumonia, poliomyelitis, chickenpox, cowpox, monkeypox, pseudorabies, roseola infantum (human herpesvirus 6), rubella

